# METTL3 enhances NSD2 mRNA stability to reduce renal impairment and interstitial fibrosis in mice with diabetic nephropathy

**DOI:** 10.1186/s12882-022-02753-3

**Published:** 2022-03-30

**Authors:** Weiming Tang, Yilin Zhao, Hui Zhang, Ying Peng, Zhilian Rui

**Affiliations:** Department of Clinical Laboratory, Liyang People’s Hospital, No. 70, Jianshe West Road, Licheng Town, Liyang, 213300 Jiangsu, P. R. China

**Keywords:** Diabetic nephropathy, NSD2, Interstitial fibrosis, METTL3, YTHDF1

## Abstract

**Background:**

Nuclear receptor-binding SET domain protein 2 (NSD2) is a histone methyltransferase that has been demonstrated to regulate insulin secretion and glucose concentration. This study focused on the role of NSD2 in the renal impairment during diabetic nephropathy (DN).

**Methods:**

Serum NSD2 level in patients with DN was examined, and its correlations with the renal impairment-related indicators were examined. A murine model of DN was established, and mouse mesangial cells (SV40-MES-13) were treated with high-glucose (HG) to mimic a DN-like condition *in vitro*. Overexpression of NSD2 was introduced into mice or cells for *in vivo* and *in vitro* studies. The m6A level in HG-treated SV40-MES-13 cells was analyzed. METTL3 expression and its correlation with NSD2 were determined.

**Results:**

NSD2 was poorly expressed in the serum of patients with DN and was negatively correlated with the levels of fasting blood sugar (FBG), serum creatinine (SCr), serum cystatin C (S-Cys-C), the 24-h urine protein (24-h U-protein) and the urine cystatin C (U-Cys-C). NSD2 overexpression reduced the kidney weight and reduced renal impairment in mice. It also suppressed interstitial fibrosis in mouse kidney tissues and reduced fibrosis-related markers in HG-treated SV40-MES-13 cells. HG treatment reduced the m6A level in the cells. METTL3 promoted m6A modification of NDS2 mRNA and enhanced its stability by YTHDF1. METTL3 overexpression alleviated renal impairment and fibrosis *in vivo* and *in vitro*. But the protective role was blocked upon NSD2 silencing.

**Conclusion:**

This study demonstrates that METTL3 promotes NSD2 mRNA stability by YTHDF1 to alleviate progression of DN.

**Supplementary Information:**

The online version contains supplementary material available at 10.1186/s12882-022-02753-3.

## Background

Diabetic nephropathy (DN), a major complication developed in approximately one-third of diabetic patients during the disease course, is one of the crucial causes leading to end-stage renal disease (ESRD) worldwide [1–3]. In the clinical practice, the presence of microalbuminuria is an important index for the onset or development of DN [4]. The most consistent and significant symptoms in renal biopsies of patients with DN are glomerular lesions such as renal fibrosis and renal impairment caused by the high glucose condition [5, 6]. Morphological and the ultrastructural changes mainly include glomerular basement membrane (GBM) thickening, mesangial expansion, glomerular hyperfiltration and tubulointerstitial fibrosis [7, 8]. However, there has no available definitive therapy for DN or the ESRD currently, and the treatments are limited to the control of hyperglycemia, lipids and blood pressure [9, 10]. Identifying novel molecular mechanisms participating in the progression of DN is of great significance to develop new therapeutic options for this disease.

Nuclear receptor-binding SET domain protein 2 (NSD2), also termed multiple myeloma SET domain or Wolf-Hirschhorn syndrome candidate 1, is a member of the SET histone methyltransferase family along with NSD1 and NSD3 [11]. It specifically modifies dimethylation or trimethylation of histone H3 lysine 36 (H3K36me2/me3), or dimethylation of H4K20 [12, 13]. Interestingly, NSD2 has been demonstrated to be downregulated in patients with type 2 diabetes mellitus, and NSD2 upregulation promoted the proliferation of pancreatic β cells and increased insulin secretion, leading to reduced glucose concentration [14]. As a high-glucose condition is likely to induce glomerular lesions and renal impairment, aberrant NSD2 expression is likely to correlate with DN development as well.

N6-methyladenosine (m6A) is the commonest internal modification in eukaryotic mRNA since its discovery in the 1970s [15–17]. The abundance and functions of m6A in RNA are governed by the interplay between its “writers” (methyltransferases, such as methyltransferase like 3 (METTL3) and METTL14); “erasers” (demethylases, such as fat mass- and obesity-associated protein) and “readers” (binding proteins, mainly including YT521-B homology domain family members (YTHDFs)) [18]. A high-glucose condition has been previously reported to reduce m6A abundance to affect gene expression [19]. Intriguingly, a “writer” of m6A modification, METTL3, was downregulated in the peripheral blood samples of diabetic patients [20], and METTL3 has been indicated to be necessary for the insulin secretion [21, 22]. We therefore, surmised that METTL3 possibly affects m6A methylation of NSD2 mRNA to regulate its expression in DN. This study was performed to examine the interaction between METTL3 and NSD2 mRNA and their functions in DN.

## Methods

### Clinical samples

Thirty-four patients with DN (46 ± 6.35 years old; 19 males and 15 females) treated at Liyang People’s Hospital from March 2019 to May 2020 were included into this research. Another 25 healthy individuals who underwent physical examination (43 ± 8.72 years old; 13 males and 12 females) were included as controls. This study was ratified by the Ethics Committee of Liyang People’s Hospital and adhered to the *Declaration of Helsinki*. Each respondent signed the informed consent.

The inclusion criteria of patients were as follows: (1) patients were diagnosed with diabetes: fasting blood sugar (FBG) ≥ 126 mg/dL (7.0 mmol/L) (American Diabetes Association Standards, 2013 [23]); (2), the renal biopsy examination results accorded with the diagnosis standard of DN. The following patients were excluded: (1) patients with type I diabetes; (2) patients with other diseases or complications, such as primary or secondary renal diseases, heart failure, malignant hypertension, cardiovascular accident, infection, liver dysfunction or aberrantly high alanine transaminase level; regnant and lactating women; (3) pregnant and lactating women; (4) patients with cancer and (5) patients without complete clinical information.

### Determination of renal impairment-related indicators

The FBG, serum creatinine (SCr), serum cystatin C (S-Cys-C), the 24-h urine protein (24-h U-protein) and urine cystatin C (U-Cys-C) were analyzed using an automatic biochemical analyzer (DxC700AU, Beckman Coulter, Fullerton, CA, USA).

### Reverse transcription quantitative polymerase chain reaction (RT-qPCR)

Total RNA from serum samples of patients, mouse kidney tissues, and from SV40-ES-13 cells was extracted using the TRIzol Reagent (Invitrogen, Thermo Scientific Pierce, Rockford, IL, USA). A PrimeScript™ RT reagent kit (Takara Holdings Inc., Kyoto, Japan) was used for cDNA synthesis. Gene expression was quantified using TB Green® Premix ExTaq^TM^II (Takara) on a CFX96 PCRsystem (Bio-Rad Inc., Hercules, CA, USA). Relative gene expression normalized to GAPDH was calculated by the 2^−ΔΔCt^ method. The primers are listed in Table 1.

**Table 1:** Primer sequences for RT-qPCR

Primers	Sequence (5'-3')
hsa-NSD2	Forward: TGTGTGAGCTGCCATGCTTCCA
	Reverse: TGAGCATCCTGCTGCCAGACAA
hsa- METTL3	Forward: CTATCTCCTGGCACTCGCAAGA
	Reverse: GCTTGAACCGTGCAACCACATC
mmu-NSD2	Forward: TTCCGCTGTCCTCTCCATAGCT
	Reverse: GCAATCACCGAACATCCTGCTG
mmu-METTL3	Forward: CAGTGCTACAGGATGACGGCTT
	Reverse: CCGTCCTAATGATGCGCTGCAG
mmu-YTHDF1	Forward: GCATCAGAAGGATGCAGTTCATG
	Reverse: GATGGTGGATAGTAACTGGACAG
hsa-GAPDH	Forward: GTCTCCTCTGACTTCAACAGCG
	Reverse: ACCACCCTGTTGCTGTAGCCAA
mmu-GAPDH	Forward: CATCACTGCCACCCAGAAGACTG
	Reverse: ATGCCAGTGAGCTTCCCGTTCAG

Note: *RT-qPCR* Reverse transcription-quantitative polymerase chain reaction, *hsa* homo sapiens, *mmu* mus musculus, *NSD2* Nuclear receptor-binding SET domain protein 2, *METTL3* Methyltransferase like 3, *YTHDF1* YT521-B homology domain family member 1, *GAPDH* glyceraldehyde-3-phosphate dehydrogenase

### Animal experiments

Male C57BL/6 mice (7 weeks old) procured from Vital River Co., Ltd. (Beijing, China) were fed in a 12-h dark/light cycle with free access to feed and water. This study was approved by the Animal Care and Ethics Committee of Liyang People’s Hospital and carried out in compliance with the Animals in Research: Reporting In vivo Experiments (ARRIVE) guidelines. All animal procedures were in accordance with the Guide for the Care and Use of Laboratory Animals (NIH Publication No. 85–23, revised 1996). Great efforts were made to minimize the pain of animals.

A murine model of DN was induced by the concomitant administration of unilateral nephrectomy (UN), high-fat-diet (HFD) and streptozotocin (STZ) as previously reported [24]. In short, the mice subjected UN after one week of acclimation to have the renal artery, renal vein and nephrotoxin of the left kidney ligated and resected. In general, mice underwent UN would not show significant symptoms, and the procedure would not affect the risk of developing hyperglycemia, hypertension or nephrotoxin. After two days, the mice were fed with normal feed (10% kcal from fat; Control group; n = 15) or HFD (70% kcal from HFD; HFD group; n = 90). All feed was procured from Trophic Animal Feed High-Tech Co., Ltd. (Nantong, Jiangsu, China).

After three weeks, the mice given HFD were intraperitoneally injected with STZ (100 mg/kg) to induce DN. The mice were continually fed for five weeks. Mice with DN were further assigned into six groups (n = 15 in each): DN group; adenovirus vector (AAV)-negative control (NC) group; AAV-NSD2 group; AAV-NSD2 + Glucagon group; AAV-METTL3 group and AAV-METTL3 + AAV-shRNA group after corresponding treatments with AAV-NC, AAV-NSD2, AAV-NSD2 + glucagon, AAV-METTL3 and AAV-METTL3 + AAV-shRNA, respectively.

The AAV overexpressing NSD2 (AAV-NSD2) and METTL3 (AAV-METTL3), and the vector silencing NSD2 (AAV-shRNA) were all procured from GenePharma Co., Ltd. (Shanghai, China). The empty AAV vector (AAV-NC; GenePharma) was used as control. The virus titer was 1 × 10^9^ TU/mL. Mice subjected to AAV treatment were injected with the corresponding AAV at one week after STZ injection. The AAV was injected at 20 μL each time, and the injection was conducted once a week for four weeks. The clinical dose of glucagon for human is 0.5 U/70 kg. The corresponding dose for mice (0.5/70) × 9.1 = 0.065 (U/kg), which was used to treat the mice.

At week 0, 3, 5 after STZ injection, respectively, five mice in each group were collected for analysis. The blood and urine samples were collected and centrifuged at 3,000 × *g* at 4 °C for 20 min. The supernatant was stored at -80 °C. The systolic blood pressure (SBP) of mice were detected using a mouse noninvasive blood pressure measurement system (Yuyan Instruments Co., Ltd., Shanghai, China). After that, the mice were sacrificed via intraperitoneal injection of 150 mg/kg pentobarbital sodium. The right kidney was collected and weighed. Thereafter, the kidney was cut into two halves. One half of the tissue was stored at -80 °C, and another half of the tissue was fixed with 4% paraformaldehyde (PFA) and embedded in paraffin.

### Histochemical staining

The paraffin-embedded tissues were cut into 5-μm sections. The sections were dewaxed and rehydrated. The pathological changes in kidney tissues were determined using a hematoxylin and eosin (HE) staining kit (G1120, Solarbio Science & Technology Co., Ltd., Beijing, China) or a Masson's trichrome staining kit (G1340, Solarbio) following the manufacturer’s protocol. All images were photographed using a camera-equipped light microscope (Nikon Instruments Inc., Tokyo, Japan).

### Enzyme-linked immunosorbent assay (ELISA)

Frozen mouse kidney tissue was made into homogenate (0.1 M Tris/HCl, pH 7.4, containing 0.5% Triton X-100, 5 mM β-ME, and 0.1 mg/ml PMSF) and centrifuged at 1,400 × *g* at 4℃ for 5 min to collect the supernatant. The contents of superoxide dismutase (SOD, #K335, BioVision, Milpitas, CA, USA), malondialdehyde (MDA, #K739, BioVision), interleukin (IL)-6 (ab100713, Abcam Inc., Cambridge, MA, USA), monocyte chemotactant protein-1 (MCP-1, ab100722, Abcam), and hydroxyproline (ab222941, Abcam) were determined adhering to the instructions of the ELISA kits. 

### Cell treatment

A mouse mesangial cell line SV40-MES-13 (CL-0470) was procured from Procell Life Science & Technology Co., Ltd. (Hubei, China). The cells were cultured in Dulbecco's modified Eagle's medium (DMEM; Gibco Company, Thermo Fisher Scientific) containing 10% fetal bovine serum (FBS) and 5.6 mM glucose at 37 °C with 5% CO_2_.

The pEXP-RB-Mam plasmid-based overexpressing vector-NSD2, vector-METTL3 and the control vector NC used for cell transfection were procured from RiboBio Co., Ltd. (Guangzhou, Guangdong China). Short hairpin (sh) RNAs of YTHDF1 and NSD2(sh-YTHDF1 and sh-NSD2) and the control sh-NC were procured from Genepharma. All transfection was conducted using the Lipofectamine 2000 (Thermo Fisher Scientific) according to the instruction manual. The cells cultured in DMEM containing 5.6 nM glucose were set as normal-glucose (NG) group, while those cultured in the medium containing 30 nM glucose were set as high-glucose (HG) group.

### Western blot analysis

Total protein in SV40-MES-13 cells or kidney tissues was extracted using the radio-immunoprecipitation assay cell lysis buffer (Beyotime Biotechnology Co. Ltd., Shanghai, China). After protein determination using a Pierce™ BCA kit (Thermo Fisher Scientific), an equal amount of protein was run on 10% SDS-PAGE and transferred loaded onto polyvinylidene fluoride membranes (Invitrogen). The membranes were blocked in 5% non-fat milk for 2 h and incubated with the primary antibodies anti-NSD2 (1:1,000, ab259940, Abcam), anti-Fibronectin (1:1,000, ab2413, Abcam), anti-collagen type I alpha 1 chain (COL1A1; 1:1,000, ab260043, Abcam), anti-E-cadherin (1:1,000, #3195, Cell Signaling Technology (CST), Beverly, MA, USA), anti-YTHDF1 (1:1,000, ab252346, Abcam) anti-METTL3 (1:1,000, ab195352, Abcam) and the internal control anti-GAPDH (1:1,000, #5174, CST) at 4℃ overnight, followed by incubation with horseradish peroxidase (HRP)-conjugated IgG (1:1,000, ab6721, Abcam) at 25℃ for 2 h. The protein signals were developed using the Pierce™ ECL system (Thermo Fisher Scientific). Relative protein level was examined using the Image J.

### 5-ethynyl-2’-deoxyuridine (EdU) labeling assay

Proliferation of the cells was examined using a BeyoClick™ EdU-647 kit (Beyotime). In short, the cells were sorted in 6-well plates. After adherence, the medium was renewed, and each well was loaded with 10 μM EdU solution and the cells were incubated at 37℃ for 2.5 h. Next, the cells were fixed in 4% PFA (Beyotime) for 15 min, permeabilized in 0.3% Triton X-100 (Elabscience Biotechnology Co., Ltd., Wuhan, Hubei, China) for 8 min, and then added with 500 μL Apollo staining buffer in the dark for 40 min. The nuclei were stained with 4', 6-diamidino-2-phenylindole (DAPI) for 10 min. The staining was observed under a fluorescence microscope (Zeiss, Oberkochen, Germany) and quantified using the Image J software.

### Immunofluorescence staining

The cells were fixed in 4% PFA for 10 min and incubated in 1% BSA/10% normal goat serum/0.3 M glycine-contained 0.1% PBS-Tween for 1 h to permeate the cells and block the non-specific protein–protein interaction. After that, the cells were incubated with anti-Fibronectin (1:250, ab2413, Abcam), anti-COL1A1 (1:250, ab270993, Abcam) and anti-E-cadherin (1:50, #3195, CST) at 4℃ overnight and then with fluorescence-conjugated IgG H&L (Alexa Fluor® 488; 1:500, ab150077, Abcam) or IgG H&L (Alexa Fluor® 647; 1:500, ab150079, Abcam) at 25℃ for 2 h. The nuclei were stained with DAPI (blue). The cells were observed under the fluorescence microscope. The mean fluorescence intensity (MFI) of proteins was analyzed using the Image J software.

### m6A quantification

Total m6A RNA methylation was determined using an EpiQuik kit (Epigentek, NY, USA) according to the manufacturer’s protocol. Total RNA was isolated using the TRIzol reagent as well. Each well was loaded with 80 μL binding solution. The RNA samples, positive control and negative control were loaded into the designative wells and allowed to stand at 37 °C for 90 min. After being washed in washing buffer for three times, the samples were incubated with 50 μL diluted capture antibody at 22℃ for 60 min, and with 50 μL detection antibody and 50 μL diluted enhancer solution for 30 min. After that, 100 μL developer solution was further added for a 10-min incubation to produce color change. The reaction was terminated by 100 μL stop solution. The optical density at 450 nm was read using a microplate reader. The m6A RNA quantity was evaluated according to the standard curve. The ratio of the amount of m6A RNA methylation to the amount of total input RNA was examined.

### m6A methylated RNA immunoprecipitation-qPCR (MeRIP-qPCR)

Total RNA from cells was extracted using a miRNeasy Mini kit (Qiagen GmbH, Hilden, Germany) through DNA digestion using the RNase-free DNase Set (Qiagen). Magnetic beads (Millipore Corp., Billerica, MA, USA) were coated with 5 μg anti-m6A (ab208577, Abcam) or anti-IgG (Abcam) at 22℃ for 30 min. The antibody-coated beads were incubated with 50 µg total RNA in RNase-inhibiting IP buffer at 4 °C overnight. After digestion in proteinase K, the m6A-bound RNA was precipitated by the phenol–chloroform RNA extraction method. The abundance of m6A was examined by RT-qPCR.

### RNA-binding protein immunoprecipitation (RIP)-qPCR

RIP was performed according to the instructions of a Magna RIP™ kit (Millipore). The magnet beads were coated with 5 μg primary antibodies (anti-YTHDF1, anti-YTHDF2, anti-YTHDF3 or anti-IgG; all acquired from Abcam) and incubated at 22℃ for 30 min. The antibody-coated magnet beads were cultured with the cells at 4 °C overnight. Then, the magnet-protein-RNA complexes were washed with RIP washing buffer for six times and then incubated with proteinase K digestion buffer at 55 °C for 30 min. The RNA was extracted using the phenol–chloroform extraction method. Then, the abundance of NSD2 was determined by RT-qPCR.

### mRNA stability analysis

The cells were treated with 5 µg/mL actinomycin D (Sigma-Aldrich Chemical Company, St Louis, MO, USA) to suppress the transcription of whole mRNA. After incubation for indicated time, the cells were collected, and the RNA sample was collected for RT-qPCR to examine the transcription level of NSD2.

### Statistical analysis

Prism 8.02 (GraphPad, La Jolla, CA, USA) was applied for data analysis. Data from at least three independent experiments were exhibited as the mean ± standard deviation (SD). Differences were analyzed by the *t* test (two groups) or one- or two-way analysis of variance (ANOVA) (multiple groups) followed by Tukey’s post-hoc test. Correlations between variables were analyzed by Pearson’s correlation analysis. *p* < 0.05 was set as the cut-off value for significant difference.

## Results

### NSD2 is poorly expressed in the patients with DN

**Fig. 1:**
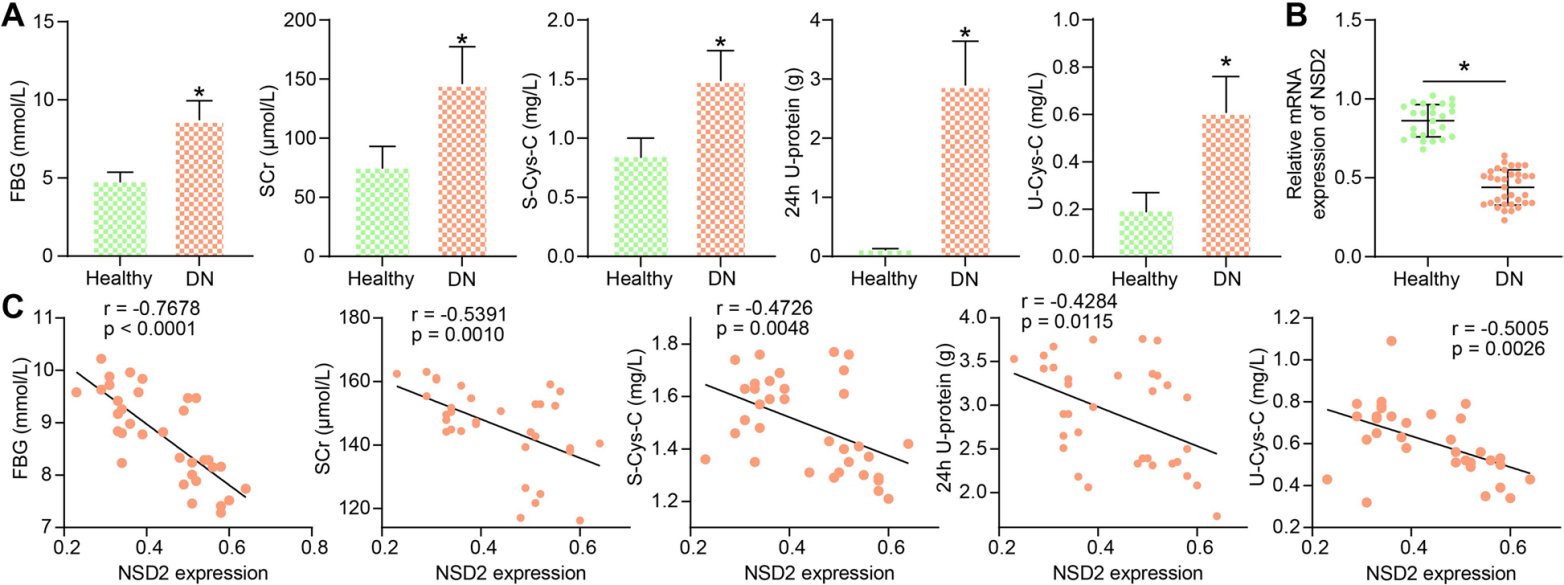
NSD2 is poorly expressed in patients with DN. **A** serum levels of FBG, SCr, S-Cys-C, 24-h U-protein, and U-Cys-C in patients with DN and in healthy individuals determined using an automatic biochemical analyzer; **B** serum level of NSD2 in patients with DN and in healthy controls determined by RT-qPCR; **C** correlations between NSD2 and the levels of FBG, SCr, S-Cys-C, 24-h U-protein, and U-Cys-C in patients with DN. There were 25 healthy individuals and 34 patients. Data were collected from three independent experiments and expressed as the mean ± SD. Differences were analyzed by the unpaired *t* test (**A**-**B**). In panel **C**, the correlations were analyzed by Pearson’s correlation analysis. *p < 0.05

The FBG, SCr, S-Cys-C, 24-h U-protein, and U-Cys-C levels in the serum samples of the patients were examined. Compared to the healthy controls, the serum levels of FBG, SCr, S-Cys-C, 24-h U-protein, and U-Cys-C were significantly elevated in patients with DN (Fig. 1A). Thereafter, the RT-qPCR results indicated that the expression of NSD2 in serum samples of patients with DN was significantly reduced (Fig. 1B). The Pearson’s correlation analyses showed that the serum NSD2 level was inversely correlated with the FBG, SCr, S-Cys-C, 24-h U-protein, and U-Cys-C levels in patients with DN (Fig. 1C).

### Overexpression of NSD2 alleviates interstitial fibrosis in the kidney tissues of mice with DN

A murine model of DN was established. The model mice were administrated with AAV-NSD2 overexpressing NSD2 to examine the function of NSD2 in DN. Since NSD2 has been reported to participate in beta-cell proliferation and result in decreased glucose concentration [14], we therefore further treated the AAV-NSD2-treated mice with glucagon to evaluate whether the effect of NSD2 on DN is glucose-dependent.

**Fig. 2:**
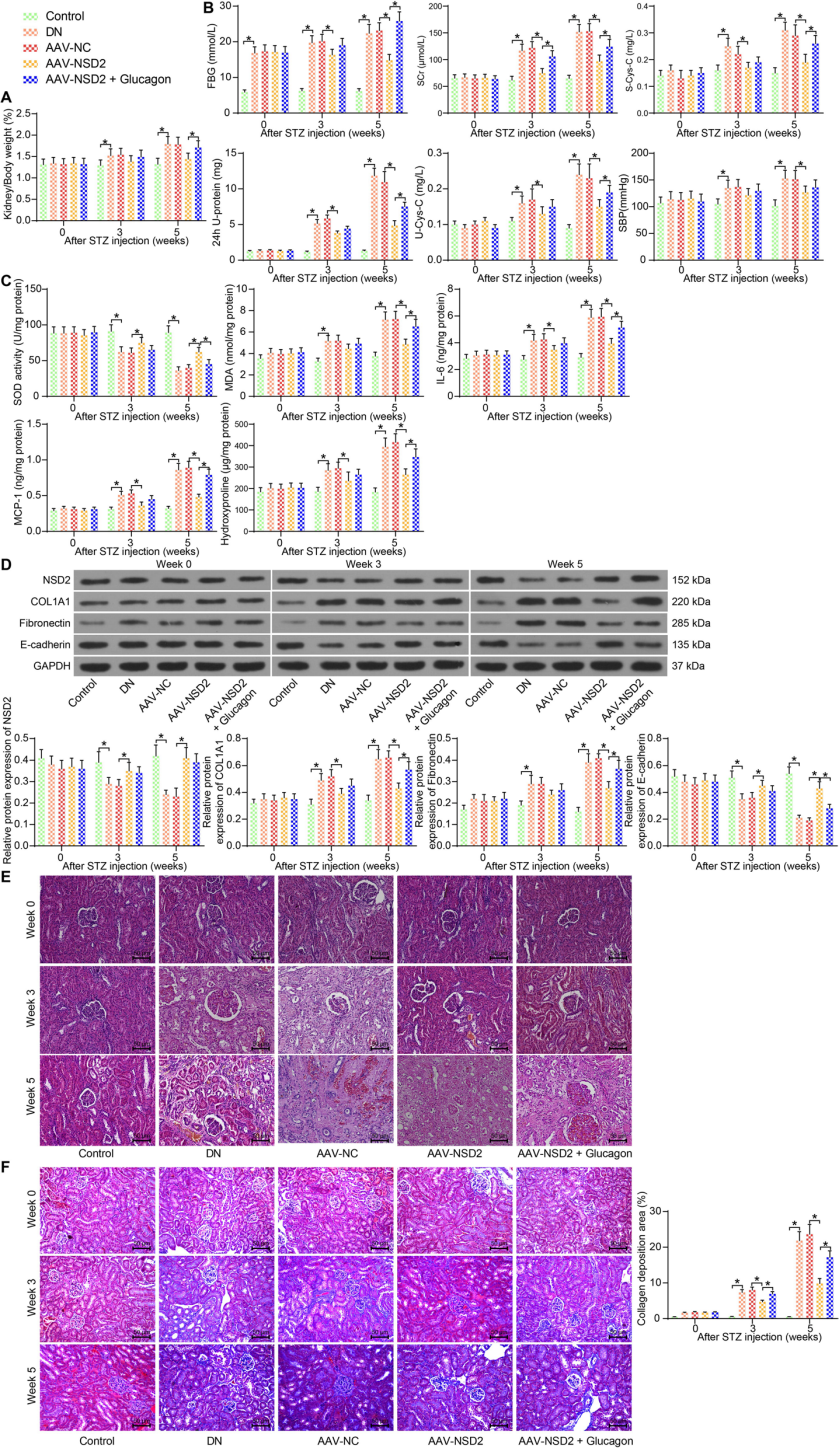
Overexpression of NSD2 alleviates interstitial fibrosis in the kidney tissues of mice with DN. **A** Kidney/body weight of mice; **B** Levels of FBG, SCr, S-Cys-C, 24-h U-protein, U-Cys-C and SBP in mice; **C** levels of SOD, MDA, IL-6, MCP-1, and hydroxyproline in mouse kidney tissues determined by ELISA kits; **D** NSD2 and the interstitial fibrosis-related proteins COL1A1, Fibronectin and E-cadherin determined by western blot analysis; **E** Pathological changes in mouse kidney tissues determined by HE staining; **F** Collagen deposition in mouse kidney tissues determined by Masson’s trichrome staining. There were at least five mice in each group. Data were collected from three independent experiments and expressed as the mean ± SD. Differences were analyzed by two-way ANOVA (**A**, **B**, **C**, **D** and **F**). *p < 0.05

The kidney/body weight and the changes of physiological and pathological indicators were analyzed. Compared to the control mice, the kidney/body weight of DN mice was significantly enhanced with time (Fig. 2A), along with incrementally increased FBG levels and SBP as well as renal impairment-related indicators SCr, S-Cys-C, 24-h U-protein, and U-Cys-C (Fig. 2B). Importantly, AAV-NSD2 treatment significantly reduced the kidney/body weight of mice and decreased the levels of FBG, SBP, SCr, S-Cys-C, 24-h U-protein, and U-Cys-C. The additional glucagon treatment significantly blocked the suppressive effects of AAV-NSD2 on FBG levels, and it also partly counteracted the suppressive effects of AAV-NSD2 on SBP, SCr, S-Cys-C, 24-h U-protein, and U-Cys-C (Fig. 2A and B).

Meanwhile, the levels of oxidative stress- and inflammation-related markers (SOD and MDA; IL-6 and MCP-1), and the contents of collagenous tissue-specific amino acid hydroxyproline in mouse kidney were determined using ELISA kits. We found that the SOD contents were decreased whereas the MDA, IL-6, MCP-1, and hydroxyproline levels were significantly elevated in DN mice. It was observed that the AAV-NSD2 treatment significantly elevated the SOD levels and suppressed the MDA, IL-6, MCP-1, and hydroxyproline levels in mouse kidneys, whereas the effect of AAV-NSD2 was partially diminished by glucagon (Fig. 2C). Moreover, the levels of NSD2 and the interstitial fibrosis-related proteins COL1A1, Fibronectin and E-cadherin were examined using western blot analysis. It was found that the levels of COL1A1 and Fibronectin in mouse kidney tissues were incrementally elevated whereas the levels of NSD2 and E-cadherin were declined in DN mice. Further AAV-NSD2 treatment significantly elevated the NSD2 and E-cadherin levels but reduced the COL1A1 and Fibronectin levels in mice. The glucagon treatment slightly counteracted the effects of AAV-NSD2, though, there showed no statistical significance (Fig. 2D). In addition, the HE staining results indicated that the mice with DN showed significant pathological symptoms, such as glomerular dilatation, glomerulosclerosis and diffuse mesangial proliferation. But the pathological changes were alleviated upon NSD2 overexpression, and the glucagon treatment blocked the effect of NSD2 in a certain extent (Fig. 2E). The Masson’s trichrome staining showed that the collagen deposition area in kidney tissues was incrementally increased in DN mice. Overexpression of NSD2 significantly decreased the collagen deposition and reduced fibrosis. However, the anti-fibrotic role of NSD2 was partially blocked by glucagon as well (Fig. 2F). Collectively, it can be inferred that NSD2 can alleviate interstitial fibrosis in the kidney tissues of mice with DN, which is partially attributed to its regulation on glucose suppression.

### NSD2 suppresses HG-induced activation of SV40-MES-13 cells and the fibrosis activity

**Fig. 3:**
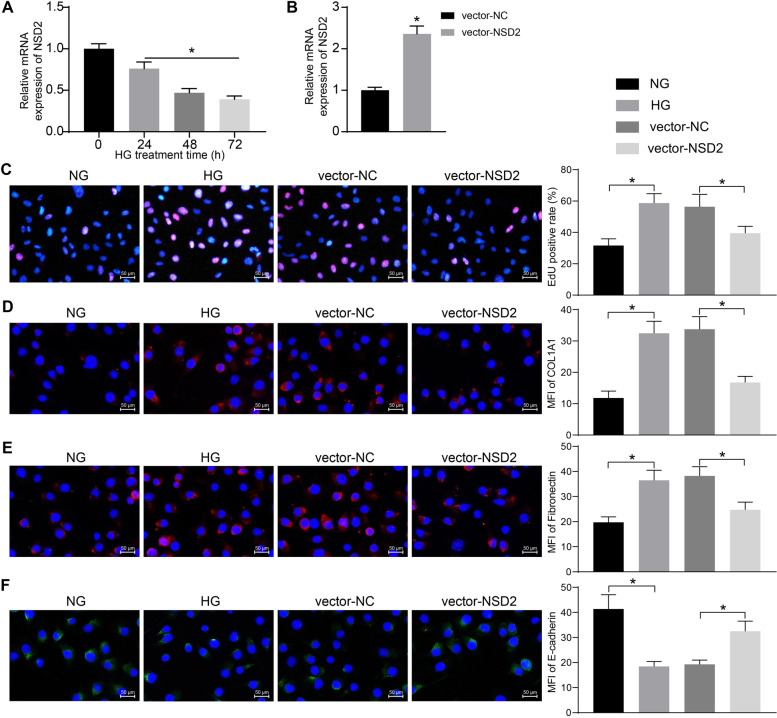
NSD2 suppresses HG-induced activation of SV40-MES-13 cells and the fibrosis activity. **A** Expression of NSD2 in SV40-MES-13 cells after HG induction determined by RT-qPCR; **B** Transfection efficacy of vector-NSD2 determined by RT-qPCR; **C** DNA replication ability of cells examined by EdU labeling; D-F, MFI of COL1A1 (**D**), Fibronectin (**E**) and E-cadherin (**F**) in cells examined by immunofluorescence staining. Data were expressed as the mean ± SD from three independent experiments. Differences were analyzed by the unpaired *t* test (**B**) or one-way ANOVA (**A**, **C**, **D**, **E** and **F**). *p < 0.05

SV40-MES-13 cells were treated with HG to mimic a DN-like condition in vitro. The RT-qPCR showed that the NSD2 expression in cells was decreased after HG treatment in a time-dependent manner (Fig. 3A). After 48 h, the NSD2 expression was declined by half. Therefore, 48 h of HG treatment was applied in the subsequent experiments.

DNA vectors overexpressing NSD2 were transfected into SV40-MES-13 cells, and the successful transfection was confirmed by RT-qPCR (Fig. 3B). Thereafter, the cells were treated with HG. EdU labeling was performed to examine proliferation activity of cells. It was found that the DNA replication ability of SV40-MES-13 cells was significantly enhanced by HG treatment but reduced upon NSD2 overexpression (Fig. 3C). Moreover, the immunofluorescence staining results indicated that HG treatment significantly reduced the expression of E-cadherin but elevated the expression of COL1A1 and Fibronectin. But these changes were prevented by NSD2 treatment (Fig. 3D-F).

### METTL3 promotes NSD2 expression

**Fig. 4:**
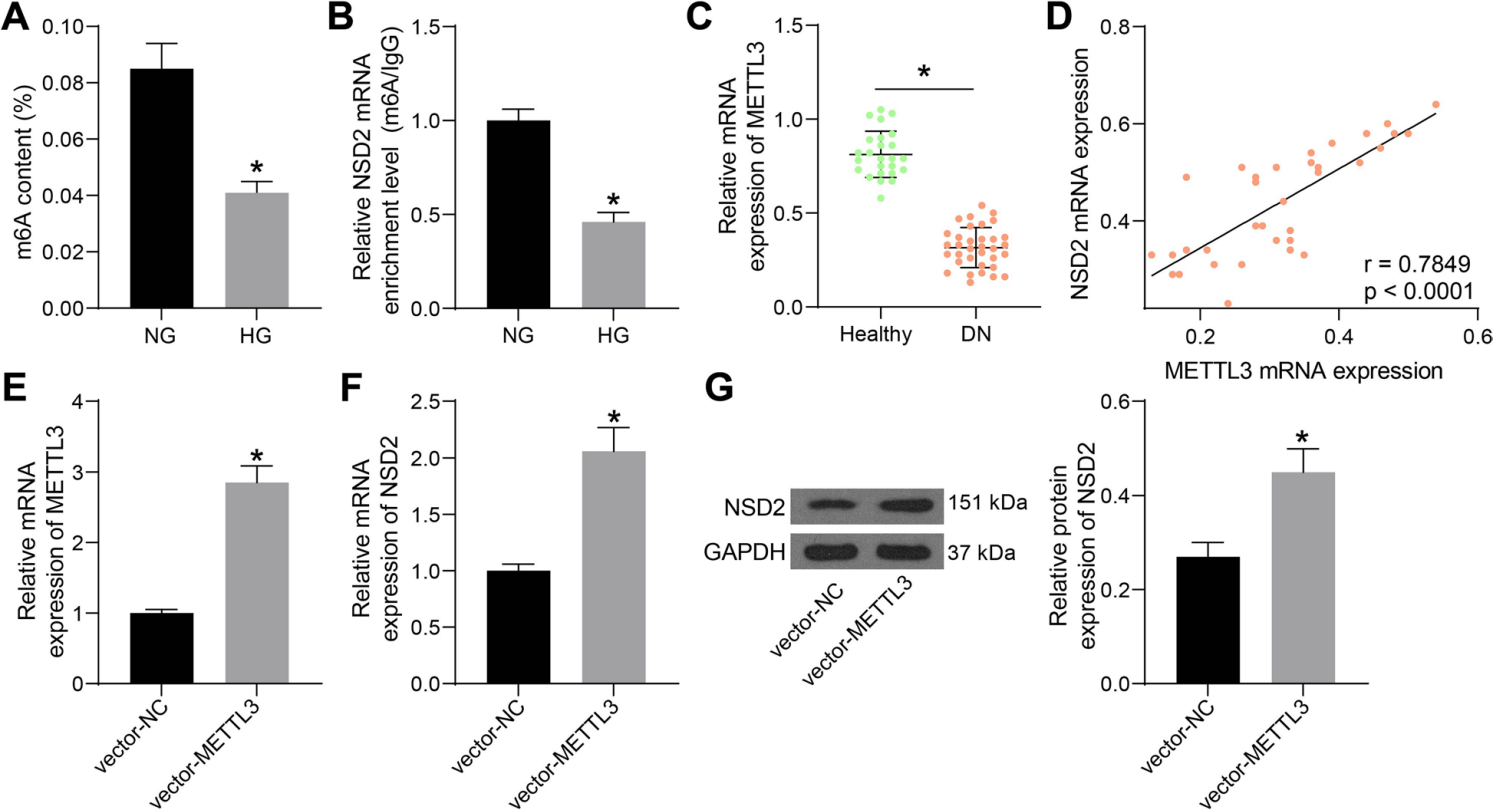
METTL3 promotes NSD2 expression. **A** m6A modification level in HG-induced SV40-MES-13 cells; **B** m6A modification on NSD2 mRNA in HG-induced SV40-MES-13 cells; **C** expression of METTL3 mRNA in patients with DN and healthy individuals examined by RT-qPCR; **D** A positive correlation between serum NSD2 and METTL3 levels in patients with DN; **E** Transfection efficacy of vector-METTL3 examined by RT-qPCR; **F**-**G**, mRNA (**F**) and protein (**G**) expression of NSD2 in cells after METTL3 overexpression determined by RT-qPCR and western blot analysis. Data were expressed as the mean ± SD from three independent experiments. Differences were analyzed by the unpaired *t* test (**A**, **B**, **C**, **E**, **F** and **G**). In panel **D**, correlation between NSD2 and METTL3 levels was analyzed by Pearson’s correlation analysis. *p < 0.05

The upstream mechanism responsible for HG-induced NSD2 downregulation was explored. HG treatment has been previously reported to reduce m6A modification to affect gene expression [19]. According to the m6A quantification assay, the m6A modification level in HG-treated SV40-MES-13 cells was significantly reduced (Fig. 4A). In addition, the subsequent MeRIP-qPCR assay indicated that HG treatment significantly reduced m6A modification of NSD2 mRNA (Fig. 4B), which might be the cause of NSD2 downregulation in the HG-treated cells.

As a “writer” of m6A modification, the expression of METTL3, which is necessary for the insulin secretion, was suggested to be reduced in the peripheral blood samples of diabetic patients [20]. We then explored the METTL3 expression in the serum samples of the patients with DN. The RT-qPCR suggested that the serum level of METTL3 was significantly reduced in patients with DN (Fig. 4C), which presented a positive correlation with the NSD2 expression (Fig. 4D). We therefore speculated that METTL3 downregulation in DN reduces m6A modification of NSD2 mRNA to induce NSD2 downregulation. Thereafter, overexpression vector of METTL3 was transfected into SV40-MES-13 cells, and the successful upregulation of METTL3 was examined by RT-qPCR again (Fig. 4E). Thereafter, it was found that METTL3 overexpression significantly elevated the expression of NSD2 in the HG-treated SV40-MES-13 cells (Fig. 4F-G).

### METTL3-YTHDF1 regulates the stability of NSD2 mRNA

**Fig. 5:**
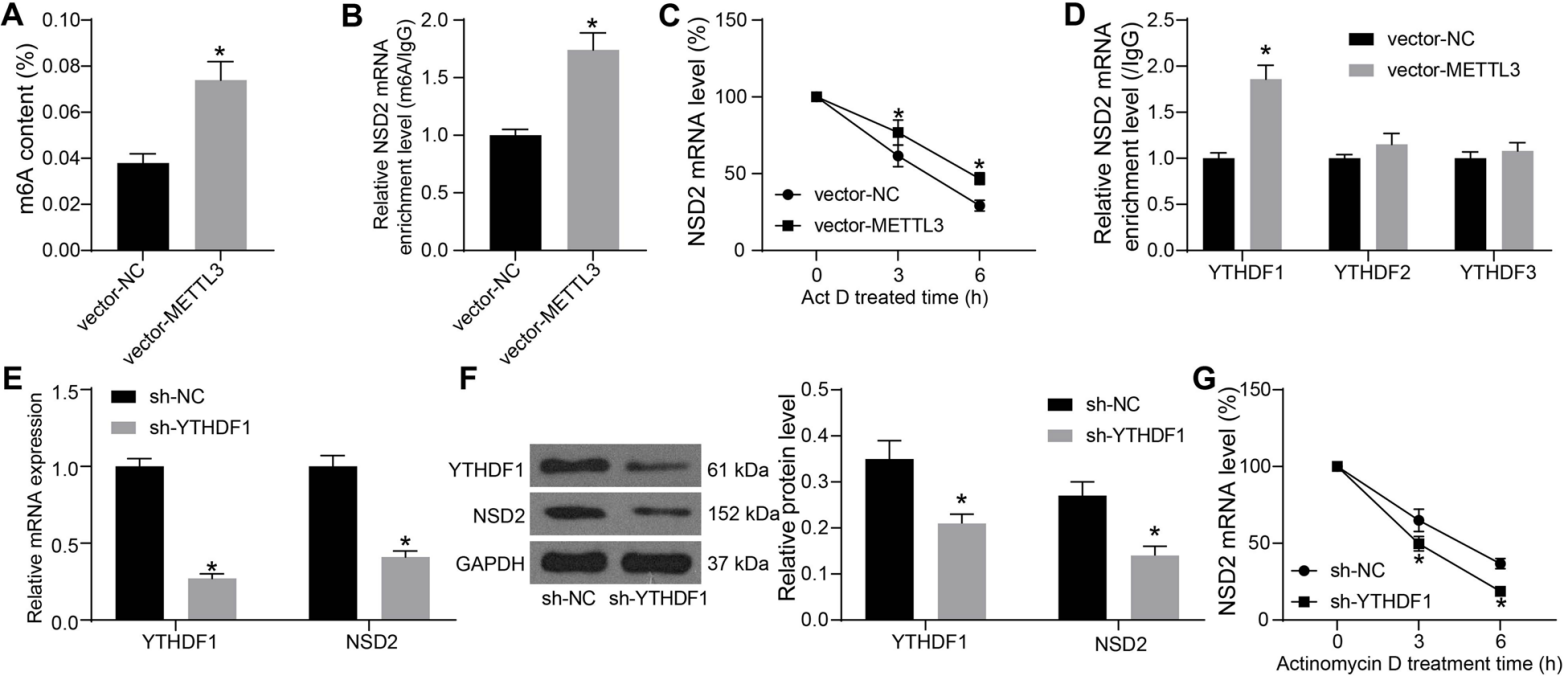
METTL3-YTHDF1 regulates the stability of NSD2 mRNA. **A** m6A level in SV40-MES-13 cells with METTL3 overexpression; **B** m6A modification level on NSD2 mRNA in SV40-MES-13 cells with METTL3 overexpression; **C** NSD2 mRNA stability in SV40-MES-13 cells with METTL3 overexpression; **D** Abundance of NSD2 mRNA enriched by YTHDF antibodies examined by RIP-qPCR; E–F, mRNA (**E**) and protein (**F**) levels of YTHDF1 and NSD2 in cells after YTHDF1 silencing examined by RT-qPCR and western blot analysis; **G**, NSD2 mRNA stability in SV40-MES-13 cells with YTHDF1 inhibition. Data were expressed as the mean ± SD from three independent experiments. Differences were analyzed by the unpaired *t* test (**A**-**B**) or two-way ANOVA (**C**-**G**). *p < 0.05

The m6A level in SV40-MES-13 cells was further examined after METTL3 overexpression. It was found that METTL3 overexpression significantly restored the m6A level in cells blocked by HG treatment (Fig. 5A). Overexpression of METTL3 also restored the m6A modification level on NSD2 mRNA after HG treatment (Fig. 5B). Moreover, METTL3 overexpression enhanced the NSD2 mRNA stability (Fig. 5C).

Thereafter, RIP and RT-qPCR assays were performed to examine the levels of common m6A modification “readers” (YTHDFs) on the NSD2 mRNA. It was found that METTL3 overexpression significantly elevated the YTHDF1 modification level on the NSD2 mRNA (Fig. 5D). Silencing of YTHDF1 was introduced in SV40-MES-13 cells. The transfection of sh-YTHDF1 significantly reduced the expression of NSD2 (Fig. 5E-F) and reduced the stability of NSD2 mRNA (Fig. 5G).

### METTL3 upregulates NSD2 to reduce HG-induced mesangial cell activation and interstitial fibrosis

**Fig. 6:**
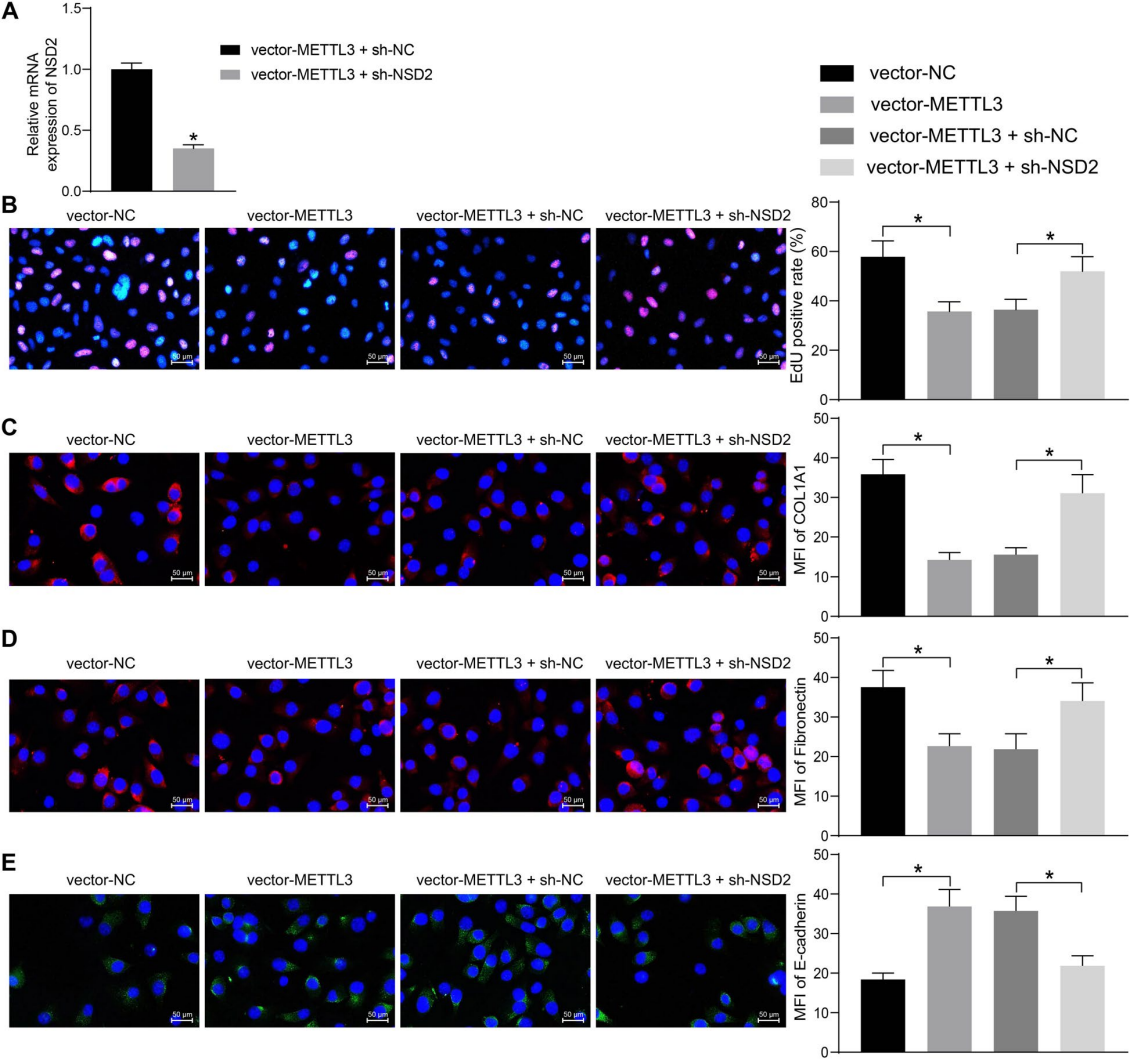
METTL3 upregulates NSD2 to reduce HG-induced mesangial cell activation and interstitial fibrosis. **A** Transfection efficacy of sh-NSD2 in SV40-MES-13 cells after vector-METTL3 transfection determined by RT-qPCR; **B** DNA replication ability of cells examined by EdU labeling; **C**-**E**, MFI of COL1A1 (**C**), Fibronectin (**D**) and E-cadherin (**E**) in cells examined by immunofluorescence staining. Data were expressed as the mean ± SD from three independent experiments. Differences were analyzed by unpaired *t* test (**A**) or one-way (**B**-**E**). *p < 0.05

To identify the interaction between METTL3 and NSD2 and their function in SV40-MES-13 cells, the cells transfected with vector-METTL3 were further transfected with shRNA of NSD2. Still, the successful transfection was confirmed by RT-qPCR (Fig. 6A). The cells were subjected to HG treatment. The EdU labeling assay suggested that the METTL3 overexpression blocked the DNA replication ability of SV40-MES-13 cells induced by HG, but that of cells was rescued when NSD2 was artificially suppressed (Fig. 6B). Likewise, the immunofluorescence staining results indicated that METTL3 overexpression led to an increase in E-cadherin expression while a decline in the expression of COL1A1 and Fibronectin. However, further silencing of NSD2 blocked the role of METTL3 and restored the expression of COL1A1 and Fibronectin whereas reduced the expression of E-cadherin (Fig. 6C-E).

### METTL3 upregulates NSD2 to alleviate interstitial fibrosis in the kidney tissues of mice with DN

**Fig. 7:**
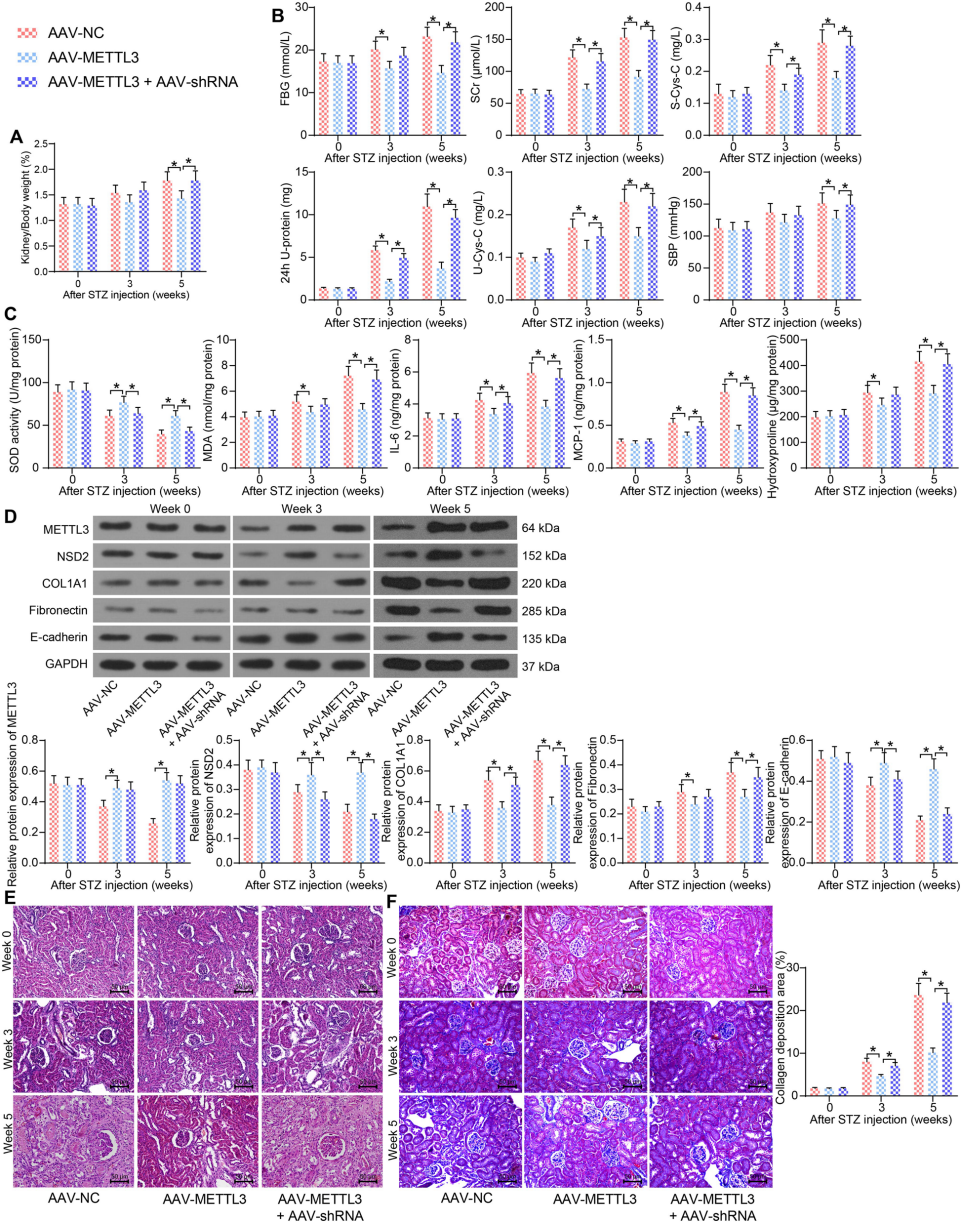
METTL3 upregulates NSD2 to alleviate interstitial fibrosis in the kidney tissues of mice with DN. **A** Kidney/body weight of mice; **B** Levels of FBG, SCr, S-Cys-C, 24-h U-protein, U-Cys-C, and SBP in mice; **C** Levels of SOD, MDA, IL-6, MCP-1, and hydroxyproline in mouse kidney tissues determined by ELISA kits; **D** Protein levels of METTL3, NSD2, and the interstitial fibrosis-related proteins COL1A1, Fibronectin and E-cadherin in mouse kidney tissues determined by western blot analysis; **E** Pathological changes in mouse kidney tissues determined by HE staining; **F** Collagen deposition in mouse kidney tissues determined by Masson’s trichrome staining. There were at least five mice in each group. Data were collected from three independent experiments and expressed as the mean ± SD. Differences were analyzed by two-way ANOVA (**A**, **B**, **C**, **D** and **F**). *p < 0.05

AAV-METTL3 overexpressing METTL3 and the AAV-shRNA silencing NSD2 were administrated into mice with DN to validate their interaction and function in vivo. Compared to AAV-NC, AAV-METTL3 significantly reduced the kidney/body weight of mice. But the concomitant administration of AAV-shRNA increased the kidney/body weight of mice with DN again (Fig. 7A). AAV-METTL3 also significantly reduced the levels of FBG, SCr, S-Cys-C, 24-h U-protein, U-Cys-C and SBP in mice, but the levels of these indicators were restored by AAV-shRNA (Fig. 7B). Moreover, the ELISA results showed that the AAV-METTL3 treatment elevated the SOD level whereas suppressed the IL-6, MCP-1 and hydroxyproline levels. Likewise, the function of AAV-METTL3 was suppressed by NSD2 silencing (Fig. 7C). In addition, the protein levels of METTL3 and NSD2 and the interstitial fibrosis-related proteins COL1A1, Fibronectin and E-cadherin in the kidney tissues were examined. The western blot analysis indicated that AAV-METTL3 treatment significantly elevated the protein levels of METTL3, NSD2 and E-cadherin in mouse kidney tissues and reduced the levels of COL1A1 and Fibronectin. As expected, further administration of AAV-shRNA reduced the levels of NSD2 and E-cadherin, but restored the levels of COL1A1 and Fibronectin in mouse kidney tissues (Fig. 7D). The HE staining results also revealed that overexpression of METTL3 mitigated the pathological changes in mice. But the DN-related symptoms in mouse kidney was aggravated again upon NSD2 silencing (Fig. 7E). The Masson’s trichrome staining suggested that the collagen deposition in mouse kidney tissues was significantly suppressed by METTL3 overexpression. However, the protective role of METTL3 was blocked by NSD2 silencing (Fig. 7F).

## Discussion

Kidneys are highly sensitive to metabolic and haemodynamic alterations, so the incidence rate of DN is expected to rise considering the increasing coverage of diabetics [25]. The DN pathogenesis has been extensively explored with no golden standard for treatment so far. Phenotypic changes of metabolites, cytokines, proteins and transcription factors are accountable for the extracellular matrix accumulation and glomerulosclerosis, namely for the progression of DN [26]. This study showed that METTL3-mediated NSD2 mRNA stability through m6A RNA methylation can alleviate renal impairment and interstitial fibrosis in DN.

Owing to its potent role in epigenetic regulation, NSD2 has been reported to play important roles in human solid tumors by regulating DNA damage repair and epithelial–mesenchymal transition process [27]. During tumor growth, reprogrammed glucose metabolism is involved to meet the demand of glycolytic intermediates for macromolecule biosynthesis, during which the NSD2 has been reported to play a role by regulating key glucose metabolism regulators, such as TIGAR, HK2, and G6PD [28]. More relevantly, NSD2 has been demonstrated to be downregulated in patients with diabetes mellitus, and its upregulation increased insulin secretion and reduced glucose concentration through promoting pancreatic β cell proliferation [14]. Moreover, NSD2-mediated H3K36 methylation has been reported to play a significant role in adipose tissue development, and NSD2 deletion was suggested to lead to lipodystrophy, which is correlated with hyperlipidemia, insulin resistance, and diabetes [29]. However, the function of NSD2 in DN has not been investigated before. Importantly, reduced expression of NSD2 was identified in patients with DN, which was correlated with increased SBP and elevated levels of FBG, SCr, Cys-C and 24-h U-protein, namely increased renal impairment. *In vivo*, overexpression of NSD2 alleviated the renal impairment and the pathological changes, including glomerular dilatation, glomerulosclerosis, diffuse mesangial proliferation, and fibrosis in mice and reduced the expression of interstitial fibrosis-related markers in tissues. *In vitro*, NSD2 overexpression also suppressed HG-induced mesangial cell (SV40-MES-13) proliferation and the expression of interstitial fibrosis-related markers as well. Collectively, these results indicated that NSD2 can mitigate renal fibrosis and impairment in DN. Interestingly, the glucagon treatment partially counteracted the functions of NSD2, indicating that the protective functions of NSD2 against DN is partially attributed to its regulation on glucose suppression.

The discovery of dynamic mRNA methylation has unveiled a new mode of post-transcriptional gene regulation in eukaryotes. Intriguingly, reduced m6A modification level, along with decreased levels of m6A-related methylases METTL3 and METTL14 were identified in HG conditions [19]. METTL3 is downregulated under the inflammatory and oxidative stress conditions and it regulates β-cell function to induce insulin secretion [21, 22]. METTL3 has been reported to be downregulated in the peripheral blood samples of diabetic patients [20]. In this study, METTL3 was identified to be significantly reduced in the serum samples of patients with DN and m6A level was reduced HG-treated SV40-MES-13 cells, while overexpression of METTL3 enhanced the NSD2 mRNA level in HG-treated cells. These results suggested that METTL3 possibly enhances NSD2 mRNA stability and expression through m6A modification. In general, METTL3 promotes m6A deposition on critical transcripts, and the subsequent mRNA translation or degradation is determined by the m6A “reader” proteins [30]. We therefore, examined the expression of several m6A “readers” YTHDFs on NSD2 mRNA, and YTHDF1 expression was found to be elevated upon METTL3 overexpression. YTHDF1 functions in promoting mRNA translation [31]. In this paper, YTHDF1 downregulation blocked the METTL3-induced NSD2 mRNA stability and NSD2 upregulation. The subsequent experiments showed that METTL3 overexpression reduced SV40-MES-13 cell proliferation and interstitial fibrosis both *in vitro* and *in vivo*, but the protective roles of METTL3 was blocked upon NSD2 silencing.

## Conclusion

It can be concluded that downregulation of NSD2 is possibly implicated in the pathogenesis and development of DN. The m6A RNA methyltransferase METTL3 can enhance NSD2 mRNA stability and expression by YTHDF1 to alleviate renal impairment and renal fibrosis in DN. Upregulation of METTL3 or NSD2 may serve as candidate therapeutic strategies for DN management. However, the effectors downstream NSD2 were not included in the present study. Moreover, we used a mesangial cell line which plays important roles in early DN for *in vitro* experiments. Validation of the interaction and roles of the METTL3/YTHDF1/NSD2 axis in other cell types such as tubular epithelial cells or mesenchymal fibroblasts is necessary to provide more comprehensive understanding of the involvement of this axis in DN. We would also like to investigate more molecules mediated by the METTL3/YTHDF1/NSD2 axis in our future researches.

## Supplementary Information

**Additional file 1.**

## Data Availability

All original data included in this study will be made available from the corresponding author upon reasonable request.

## Abbreviations

AAV: Adenovirus vector
ANOVA: Analysis of variance
COL1A1: Collagen type I alpha 1 chain
DMEM: Dulbecco's modified Eagle's medium
DN: Diabetic nephropathy
EdU labeling: 5-Ethynyl-2’-deoxyuridine
ESRD: End-stage renal disease
FBG: Fasting blood sugar; FBS, fetal bovine serum
GAPDH: Glyceraldehyde-3-phosphate dehydrogenase
GBM: Glomerular basement membrane
HE staining: Hematoxylin and eosin staining
HFD: High-fat-diet
HG: High-glucose
HRP: Horseradish peroxidase
IgG: Immunoglobulin G;
MCP-1: Monocyte chemotactant protein-1
MDA: Malondialdehyde
IL-6: Interleukin-6
mean ± SD: Mean ± standard deviation
MERIP-qPCR: M6A methylated RNA immunoprecipitation-qPCR
METTL3: Methyltransferase like 3
MFI: Mean fluorescence intensity
NC: Negative control
NG: Normal-glucose
NSD2: Nuclear receptor-binding SET domain protein 2
PFA: Paraformaldehyde
RT-qPCR: Reverse transcription quantitative polymerase chain reaction
SBP: Systolic blood pressure
SCr: Serum creatinine
S-Cys-C: Serum cystatin C
SDS-PAGE: Sodium dodecyl sulfate–polyacrylamide gel electrophoresis
shRNA: Short hairpin RNA
SOD: superoxide dismutase
STZ: Streptozotocin
U-Cys-C: Urine cystatin C
UN: Unilateral nephrectomy
YTHDFs: YT521-B homology domain family members
24-h U-protein: 24-H urine protein

## References

[1] Ibrahim HN, Hostetter TH (1997). Diabetic nephropathy. J Am Soc Nephrol.

[2] Ioannou K (2017). Diabetic nephropathy: is it always there? Assumptions, weaknesses and pitfalls in the diagnosis. Hormones (Athens).

[3] Sanajou D, GhorbaniHaghjo A, Argani H, Aslani S (2018). AGE-RAGE axis blockade in diabetic nephropathy: current status and future directions. Eur J Pharmacol.

[4] Giralt-Lopez A, Molina-Van den Bosch M, Vergara A, Garcia-Carro C, Seron D, Jacobs-Cacha C (2020). Revisiting experimental models of diabetic nephropathy. Int J Mol Sci.

[5] Maezawa Y, Takemoto M, Yokote K (2015). Cell biology of diabetic nephropathy: Roles of endothelial cells, tubulointerstitial cells and podocytes. J Diabetes Investig.

[6] Sun YM, Su Y, Li J, Wang LF (2013). Recent advances in understanding the biochemical and molecular mechanism of diabetic nephropathy. Biochem Biophys Res Commun.

[7] Gnudi L, Coward RJM, Long DA (2016). Diabetic nephropathy: perspective on novel molecular mechanisms. Trends Endocrinol Metab.

[8] Qi C, Mao X, Zhang Z, Wu H (2017). classification and differential diagnosis of diabetic nephropathy. J Diabetes Res.

[9] Flyvbjerg A (2017). The role of the complement system in diabetic nephropathy. Nat Rev Nephrol.

[10] Lu Y, Liu D, Feng Q, Liu Z (2020). diabetic nephropathy: perspective on extracellular vesicles. Front Immunol.

[11] Tanaka H, Igata T, Etoh K, Koga T, Takebayashi SI, Nakao M (2020). The NSD2/WHSC1/MMSET methyltransferase prevents cellular senescence-associated epigenomic remodeling. Aging Cell.

[12] Li Y, Trojer P, Xu CF, Cheung P, Kuo A, Drury WJ (2009). The target of the NSD family of histone lysine methyltransferases depends on the nature of the substrate. J Biol Chem.

[13] Morishita M, Mevius D, di Luccio E (2014). In vitro histone lysine methylation by NSD1, NSD2/MMSET/WHSC1 and NSD3/WHSC1L. BMC Struct Biol.

[14] Shi S, Zhao L, Zheng L (2018). NSD2 is downregulated in T2DM and promotes beta cell proliferation and insulin secretion through the transcriptionally regulation of PDX1. Mol Med Rep.

[15] Desrosiers R, Friderici K, Rottman F (1974). Identification of methylated nucleosides in messenger RNA from Novikoff hepatoma cells. Proc Natl Acad Sci U S A.

[16] Wang X, Lu Z, Gomez A, Hon GC, Yue Y, Han D (2014). N6-methyladenosine-dependent regulation of messenger RNA stability. Nature.

[17] Wei CM, Gershowitz A, Moss B (1975). Methylated nucleotides block 5' terminus of HeLa cell messenger RNA. Cell.

[18] Yang Y, Hsu PJ, Chen YS, Yang YG (2018). Dynamic transcriptomic m(6)A decoration: writers, erasers, readers and functions in RNA metabolism. Cell Res.

[19] Xu Z, Jia K, Wang H, Gao F, Zhao S, Li F (2021). METTL14-regulated PI3K/Akt signaling pathway via PTEN affects HDAC5-mediated epithelial-mesenchymal transition of renal tubular cells in diabetic kidney disease. Cell Death Dis.

[20] Zha X, Xi X, Fan X, Ma M, Zhang Y, Yang Y (2020). Overexpression of METTL3 attenuates high-glucose induced RPE cell pyroptosis by regulating miR-25-3p/PTEN/Akt signaling cascade through DGCR8. Aging (Albany NY).

[21] Li X, Jiang Y, Sun X, Wu Y, Chen Z (2021). METTL3 is required for maintaining beta-cell function. Metabolism.

[22] Wang Y, Sun J, Lin Z, Zhang W, Wang S, Wang W (2020). m(6)A mRNA methylation controls functional maturation in neonatal murine beta-cells. Diabetes.

[23] American DA (2013). Diagnosis and classification of diabetes mellitus. Diabetes Care.

[24] Kim H, Dusabimana T, Kim SR, Je J, Jeong K, Kang MC (2018). Supplementation of abelmoschus manihot ameliorates diabetic nephropathy and hepatic steatosis by activating autophagy in mice. Nutrients.

[25] Magee C, Grieve DJ, Watson CJ, Brazil DP (2017). Diabetic nephropathy: a tangled web to unweave. Cardiovasc Drugs Ther.

[26] Conserva F, Pontrelli P, Accetturo M, Gesualdo L (2013). The pathogenesis of diabetic nephropathy: focus on microRNAs and proteomics. J Nephrol.

[27] Chen R, Chen Y, Zhao W, Fang C, Zhou W, Yang X (2020). The role of methyltransferase NSD2 as a potential oncogene in human solid tumors. Onco Targets Ther.

[28] Wang J, Duan Z, Nugent Z, Zou JX, Borowsky AD, Zhang Y (2016). Reprogramming metabolism by histone methyltransferase NSD2 drives endocrine resistance via coordinated activation of pentose phosphate pathway enzymes. Cancer Lett.

[29] Zhuang L, Jang Y, Park YK, Lee JE, Jain S, Froimchuk E (2018). Depletion of Nsd2-mediated histone H3K36 methylation impairs adipose tissue development and function. Nat Commun.

[30] Zeng C, Huang W, Li Y, Weng H (2020). Roles of METTL3 in cancer: mechanisms and therapeutic targeting. J Hematol Oncol.

[31] Han B, Yan S, Wei S, Xiang J, Liu K, Chen Z (2020). YTHDF1-mediated translation amplifies Wnt-driven intestinal stemness. EMBO Rep.

