# Supplementary material for: METTL3 enhances NSD2 mRNA stability to reduce renal impairment and interstitial fibrosis in mice with diabetic nephropathy
Source: BMC Nephrol. 2022 Mar 30;23:124. doi: 10.1186/s12882-022-02753-3 (PMC8969340; doi:10.1186/s12882-022-02753-3)
Supplement: Supplementary file 1 — Additional file 1. [file 12882_2022_2753_MOESM1_ESM.docx]

**Supplementary Files of original, unprocessed images**

**Fig 2D Week 0**

**
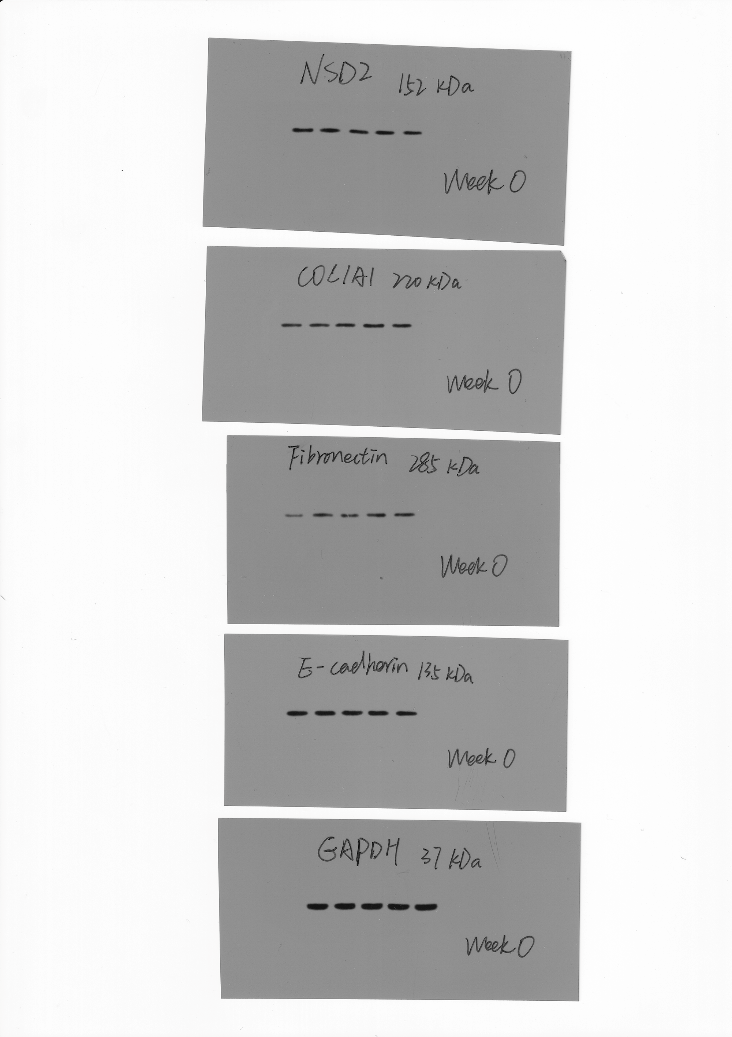
**

**Fig 2D Week 3**

**
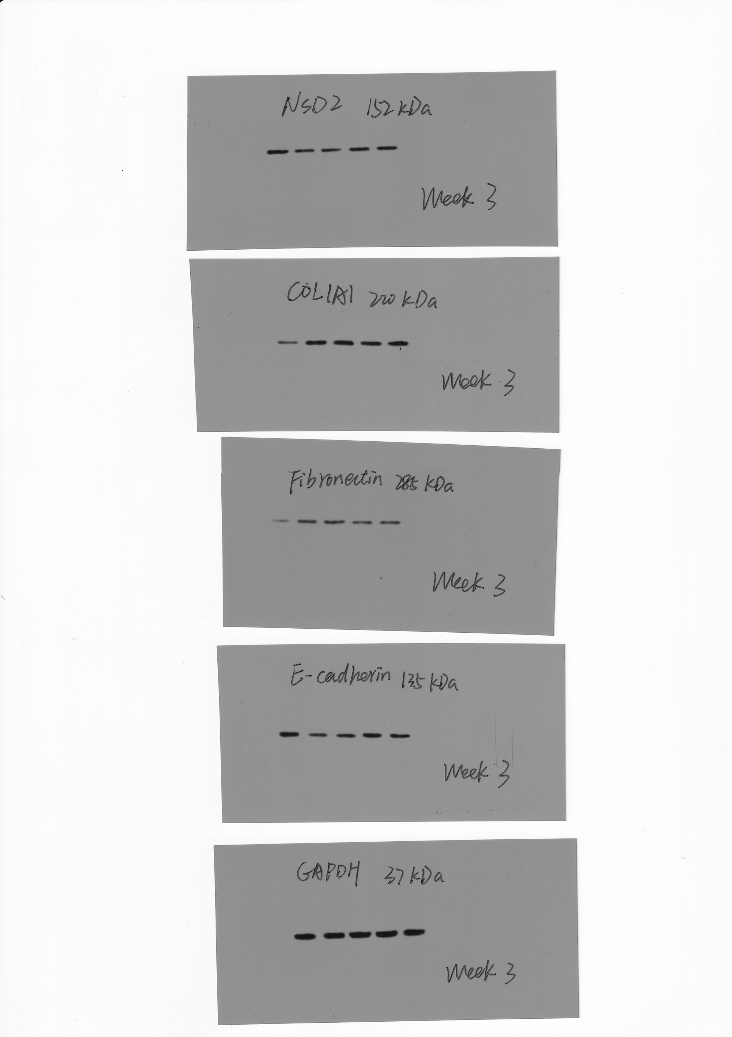
**

**Fig 2D Week 5**

**
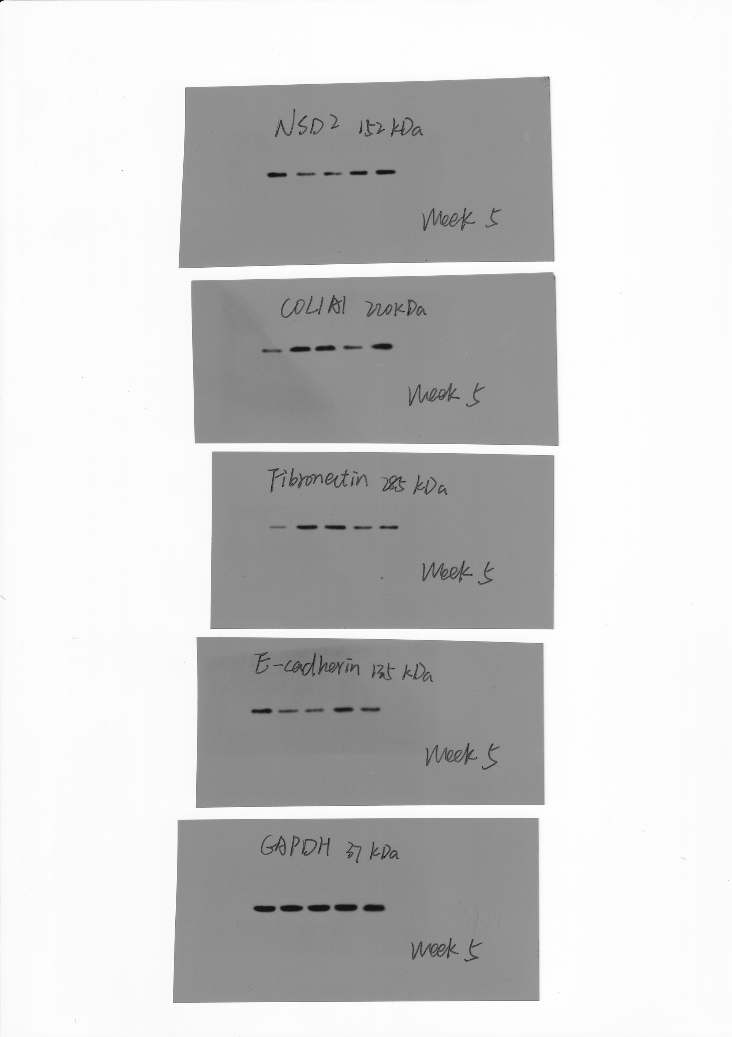
**

**Fig 4G**

**
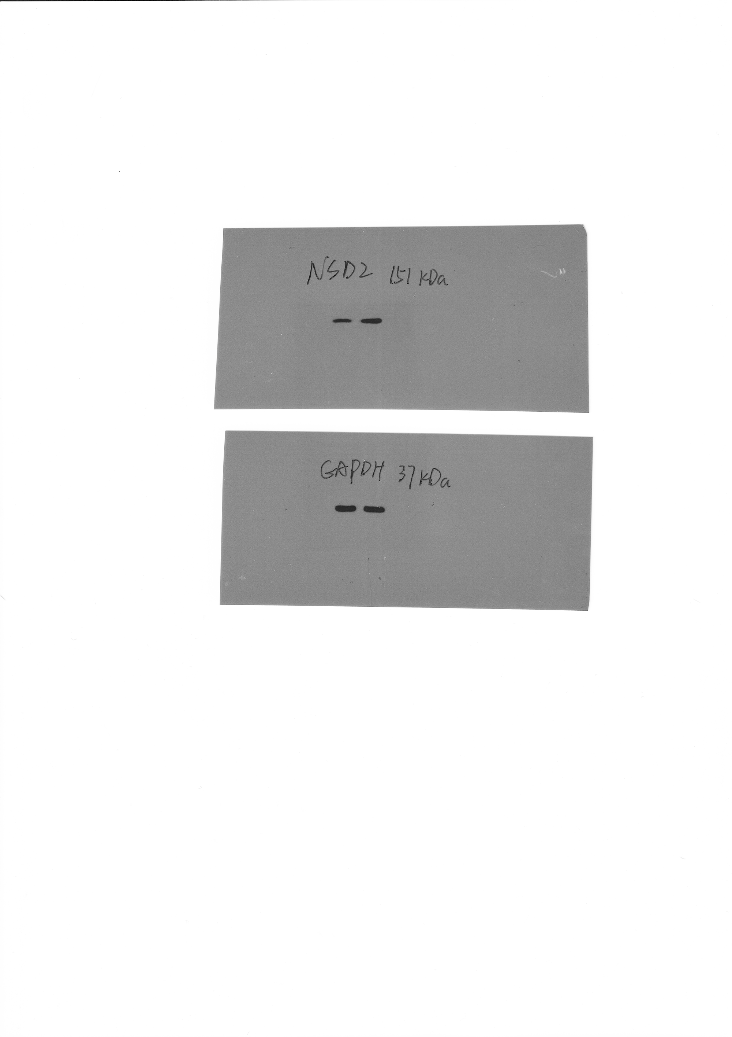
**

**Fig 5F**

**
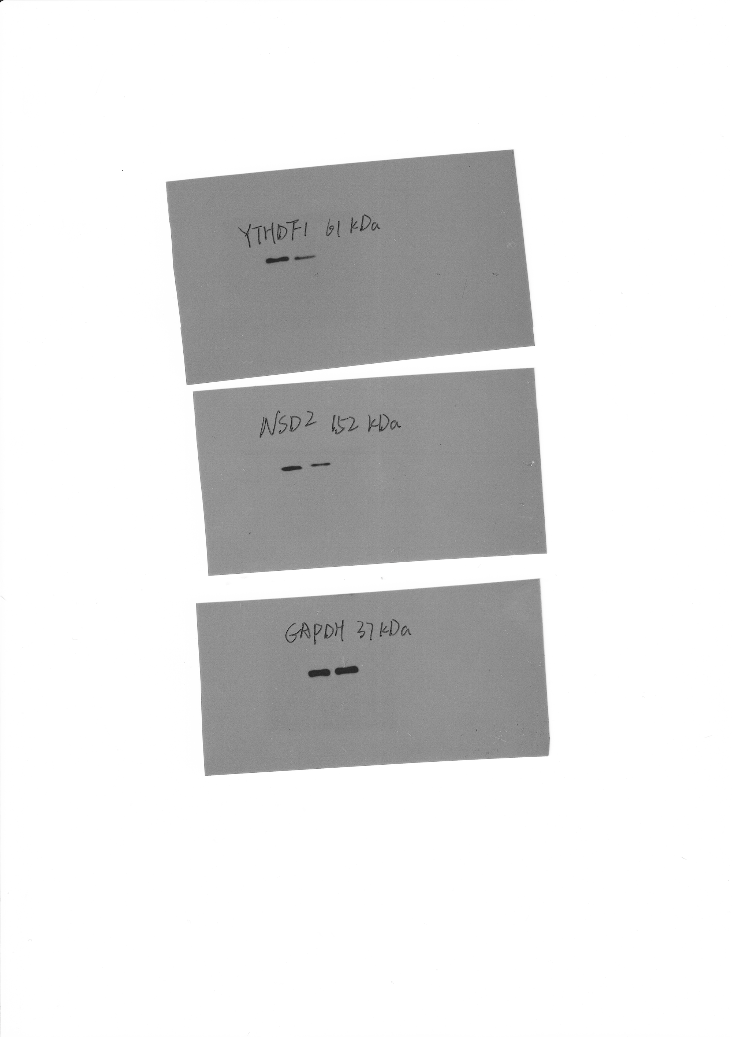
**

**Fig 7D Week 0**

**
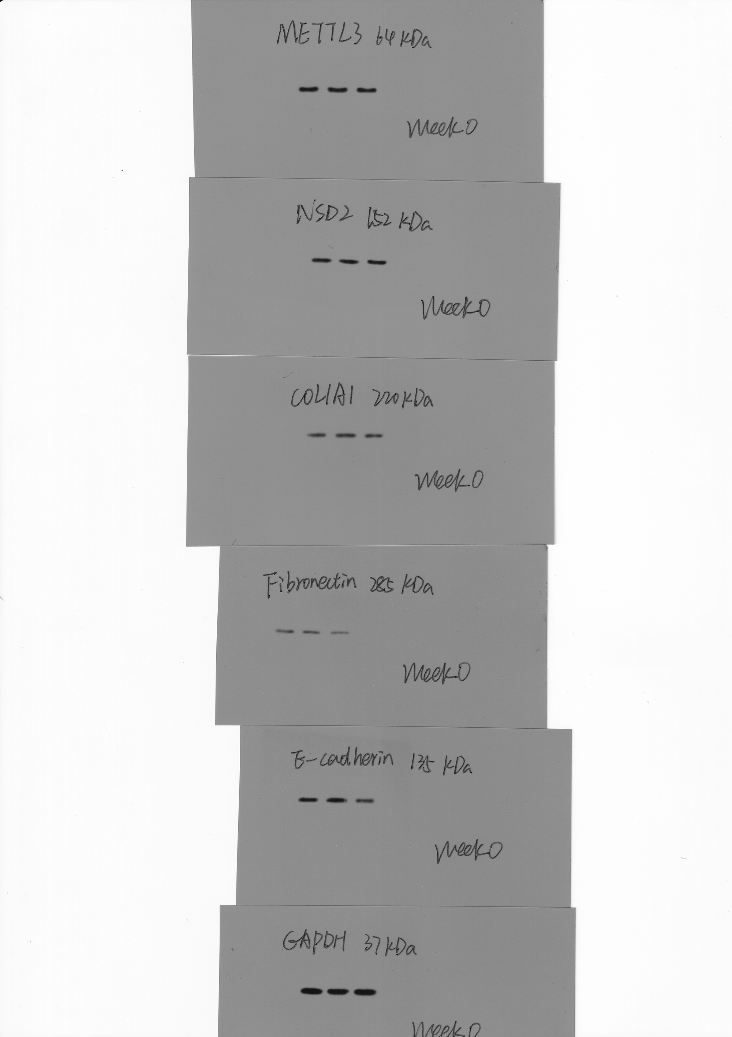
**

**Fig 7D Week 3**

**
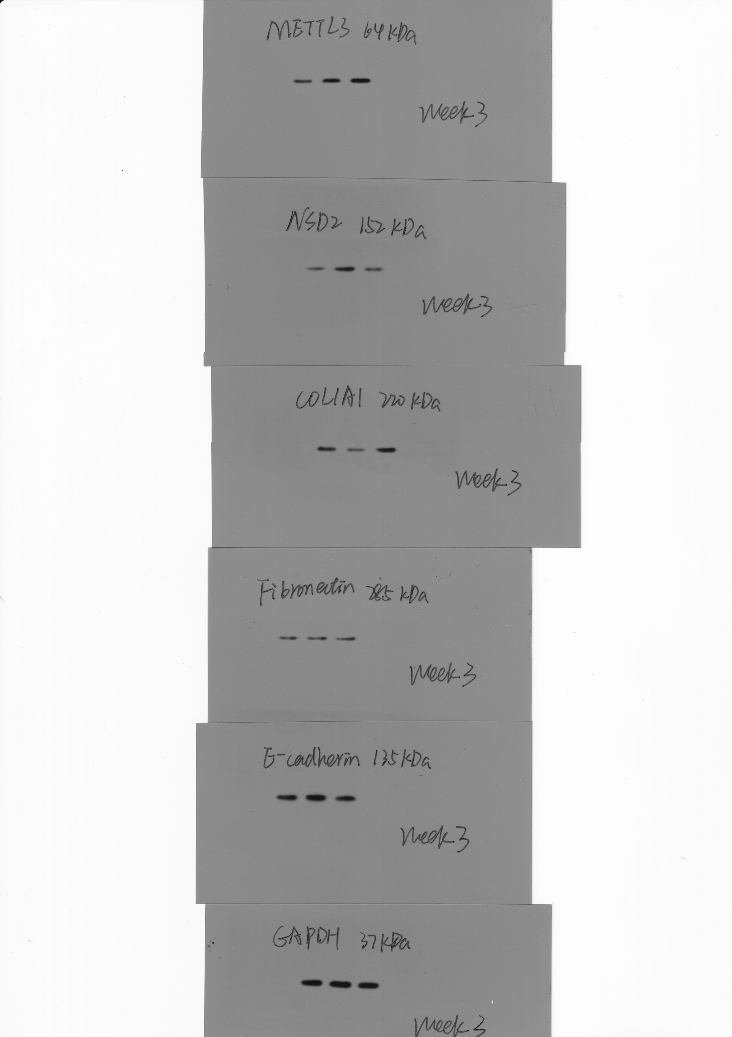
**

**Fig 7D Week 5**

**
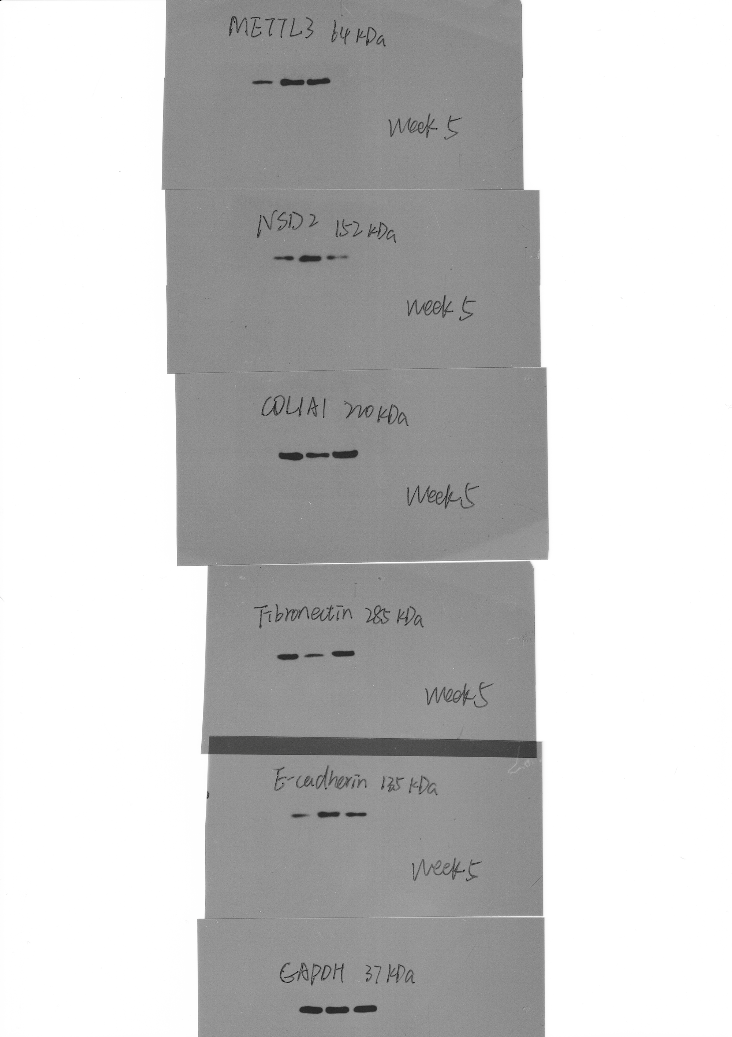
**
